# More Danger Than Meets the Eye: Potentially Toxic Element Contamination in Fish from the Western Pará Poses Significant Hazards to Local Communities

**DOI:** 10.1021/acsomega.5c10676

**Published:** 2026-02-10

**Authors:** Fábio Edir Amaral Albuquerque, Francisco Flávio Vieira de Assis, Marta Miranda, Rejane Santos Sousa, Raimundo Alves Barrêto-Júnior, Marta López Alonso, Antonio Humberto Hamad Minervino

**Affiliations:** † Laboratory of Animal Health, LARSANA, Federal University of Western Pará UFOPA, Santarém, Pará 68040-255, Brazil; ‡ Ph.D. Program in Society, Nature and Development (PPGSND), Federal University of Western Pará (UFOPA), Santarém, Pará 68040-255, Brazil; § Department of Anatomy, Animal Production and Clinical Veterinary Sciences, Veterinary Faculty, 16780 University of Santiago de Compostela, Lugo 27002, Spain; ∥ Federal University of Southern and Southeastern Pará - UNIFESSPA, Xinguara, Pará 68555-000, Brasil; ⊥ Department of Animal Science, Federal Rural University of the Semiarid, Mossoró, Rio Grande do Norte 59625-900, Brazil; # Department of Animal Pathology, Veterinary Faculty, University of Santiago de Compostela, Lugo 27002, Spain

## Abstract

The Amazon basin is undergoing rapid environmental transformation driven by agricultural expansion and mining activities, resulting in increased concentrations of toxic metals in aquatic ecosystems. This study quantified arsenic (As), cadmium (Cd), mercury (Hg), and lead (Pb) in six fish species and evaluated associated noncarcinogenic and carcinogenic health risks under two consumption scenarios: the Amazon Scenario (462 g/person/day) and the Brazil Scenario (24 g/person/day). Fish were sampled in five municipalities in western Pará, which differ in the intensity of the gold and bauxite mining activities. The results show that Hg concentrations exceeded legal limits in most carnivorous species; the target hazard quotients (THQ) indicate lifelong noncarcinogenic risk (THQ > 1) in nearly all samples under the Amazon Scenario, peaking at 28.97 for *Cichla ocellaris* from Porto Trombetas. Total target hazard quotients (TTHQs) also exceeded the safety threshold of 1 for all species in the Amazon Scenario, indicating significant noncarcinogenic risk for local consumers, whereas risks remained acceptable under the national consumption pattern. Carcinogenic risk analysis revealed that 25% of samples in the Amazon Scenario exceeded the 1 × 10^–4^ threshold, primarily due to arsenic exposure. These findings demonstrate that traditional fish-based diets expose Amazonian riverine populations to hazardous levels of potentially toxic elements, underscoring the need for integrated environmental monitoring, public health surveillance, and nutritional guidance tailored to high-consumption communities.

## Graphical abstract

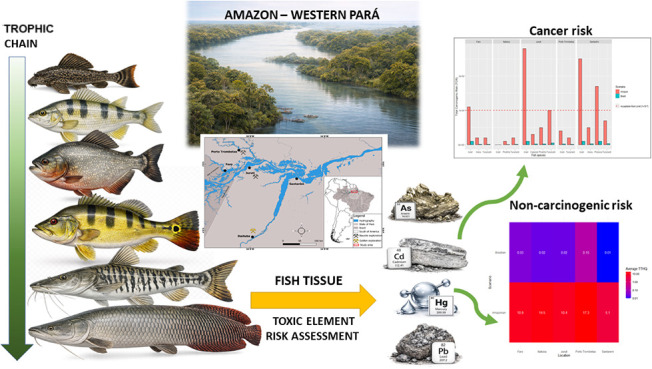

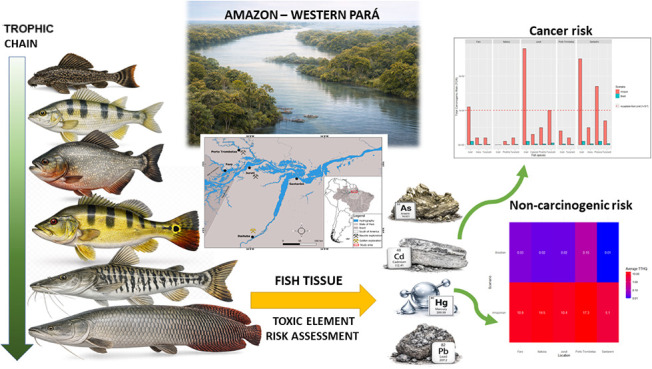

## 1 Introduction

In recent years, the western region of the Pará state has undergone significant environmental challenges. Artisanal gold mining, mainly illegal, is a well-established pollutant activity in the region and is responsible for direct Hg contamination, but in recent years, gold mining has considerably increased in the region, with a change in the mining profile as miners have started using heavy equipment, leading to greater industrialization of the process and, consequently, greater environmental damage.^1^ The region also has large mining projects, mainly bauxite, in Porto Trombetas (municipality of Oriximiná) since 1969, and in the municipality of Juruti, since 2009. Mining is a known pollutant activity, with a reported red mud spillover in Batata Lake, Porto Trombetas, between 1979 and 1989.^2–4^ Red mud, a highly alkaline waste residue from bauxite processing in aluminum production, contains elevated concentrations of heavy metals.^5^ Environmental risks arise from its high pH, which can cause soil permeation issues and groundwater contamination, leading to heavy metal leaching, high sodium content, persistent alkalinity, and elevated moisture levels, and substantial transportation and disposal costs,^6^ as well as the well-documented track record of environmental contamination by mercury in the Tapajós River basin associated with artisanal gold mining.^7–10^

In addition, the region has been subject to a rapid agricultural transformation, from smallholder farmers to industrial mechanized agriculture of corn and soybeans. The lower Amazon region, within western Pará, became a new agricultural frontier, with an increase in soybean planted area from only 25 ha in 2001 to 122,000 ha in 2024.^11^ Both this agricultural expansion and ongoing mining activities (including intensified artisanal gold mining and large-scale bauxite operations) have driven widespread direct deforestation through land clearing and the construction of extensive infrastructure networks.^12^ Since Hg is abundantly present in the soils of the region,^13^ vegetation removal and soil disturbance promote accelerated erosion that contribute to a greater availability of Hg in aquatic ecosystems.^14,15^

These anthropogenic activities have impacted local populations, who are highly dependent on rivers for their livelihoods. Freshwater fish are the main source of protein in riverside communities, where the average annual local consumption of fish is approximately 94 kg per person, almost six times the global average,^16^ reaching 169 kg per person/year in isolated communities.^17^ Despite its economic and food importance in the region, fish species can accumulate potentially toxic elements, especially inorganic metals.^18,19^ Thus, these species may represent the main sources of dietary toxic elements in areas with environmental impacts. Although only the effects of Hg have been considered in the region, little is known about the accumulation of toxic elements such as As, Cd, and Pb, which pose significant chronic health risks to humans through bioaccumulation in fish consumed by riparian communities.^20^ These risks arise from both geogenic sources^21^ and anthropogenic activities prevalent in the Amazon.^22,23^

Exposure to toxic elements varies widely among the fish species consumed in the Amazon region. Large carnivorous species at the top of the food chain accumulate considerably higher levels of Hg than other species of herbivorous fish and smaller detritivores.^18,19^ In the Amazon, the Hg levels recommended by the WHO are surpassed in most fish, mainly in carnivorous species,^24,25^ and inhabitants, who consume these fish, have Hg levels in their blood and hair that indicate toxicity.^26,27^

The riverine and indigenous communities located in the Lower Amazon and Tapajós River basins heavily depend on fishing as their main source of protein. In this region, predatory species popularly known as Tucunaré, Pintado, Pirarucu, Piranha, and the nonpredatory Aracu and acari are particularly valued. When considering the potential of fish as a regional resource, it is crucial to take into account its capacity, especially in species at the top of the food chain, to accumulate residues of toxic metals, exacerbating health risks through food exposure. The hypothesis of the study is that historical mining activities and recent agricultural expansion in the region have had significant impacts on the levels of toxic elements in the main commercially traded species, potentially exceeding USEPA safety thresholds in high-consumption scenarios (Amazon Scenario, 462 g/day), leading to elevated noncarcinogenic and carcinogenic risks for riparian populations.

This study aims to quantify As, Cd, Hg, and Pb levels in six fish species from five cities in the Western Pará State, Brazilian Amazon, and to assess noncarcinogenic and carcinogenic health risks for local populations under high (Amazon Scenario) and low (Brazil Scenario) consumption patterns.

## 2 Methods

### 2.1 Study Site and Sample Collection

Fish samples were collected from five sites in western Pará (Figure 1). These five sites, located in the municipalities of Faro, Juruti, Santarém, and Oriximiná in the Lower Amazon Mesoregion and Itaituba in the Southwest Pará Mesoregion, were selected to represent diverse environmental and anthropogenic conditions within the Amazon and Tapajós river basins.

**Figure 1:**
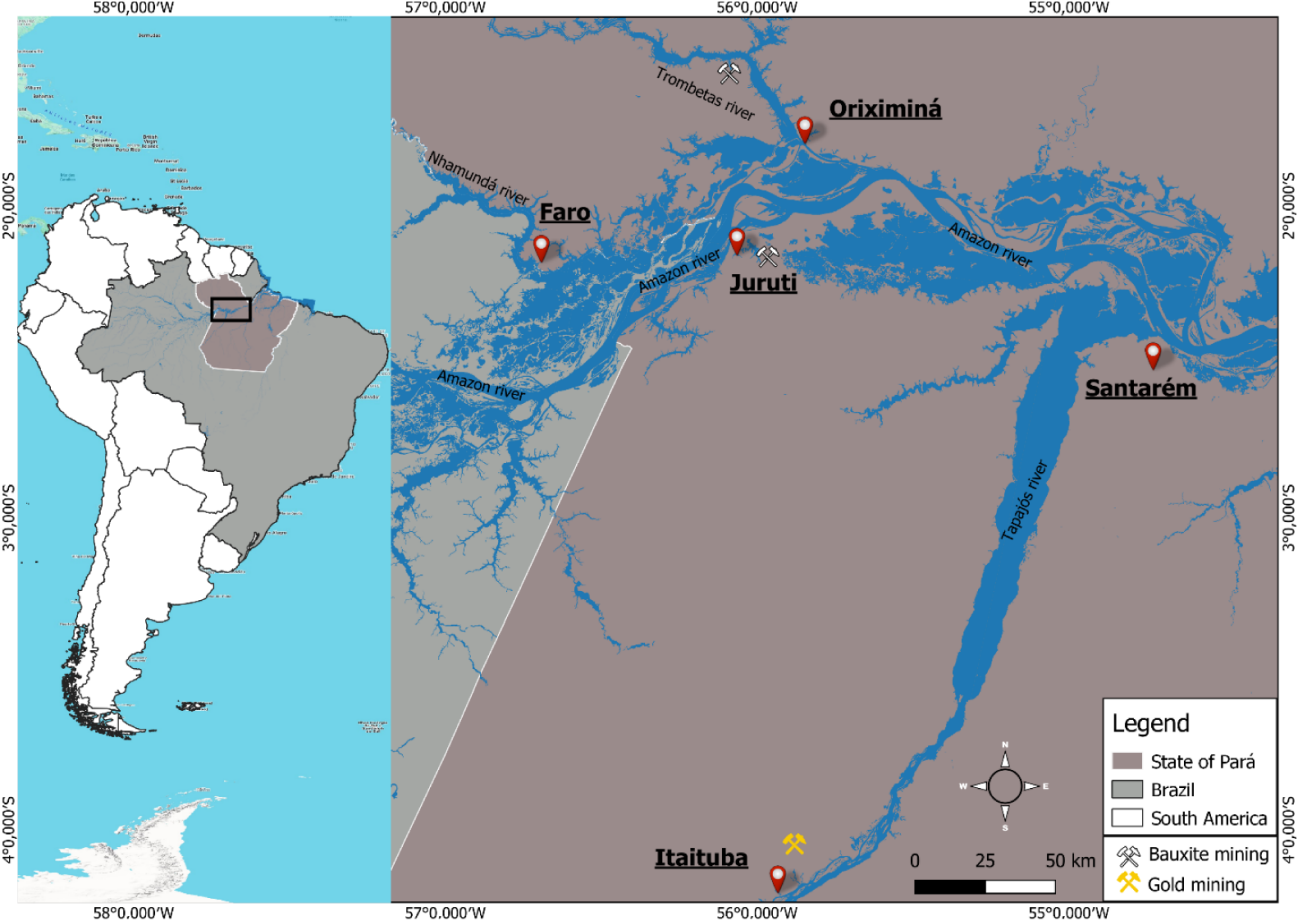
Identification and location of sampling sites in the western region of the state of Pará. The symbols on the map indicating gold and bauxite mining do not have a precise geographic location, since there are several small-scale artisanal mining activities in the region.

Faro (1), located at the confluence of the Nhamundá and Amazon rivers, has floodplain ecosystems and no mining activity, serving as a reference for initial levels of potentially toxic elements in fish; Juruti (2), with bauxite mining initiated 16 years ago; Santarém (3), the largest urban center, with more than 350,000 inhabitants,^28^ is located at the confluence of the Amazon and Tapajós rivers, downstream of mining areas, and has undergone intense deforestation due to agricultural expansion. Porto Trombetas (4), in the municipality of Oriximiná, is located on the banks of the Trombetas River, where bauxite mining since 1969 may contribute to heavy metal pollution, particularly As and Cd, from red mud residues; Itaituba (5), on the Tapajós River, is a gold mining hub, a major source of mercury contamination.

To ensure sample authenticity and address the uncertainties often associated with indirect collection methods, such as purchasing fish from markets without precise knowledge of their origin, we employed a distinctive approach by collecting fish directly during fishing activities in collaboration with local fishermen. A total of 398 specimens from six fish species (Table 1) were sampled: one detritivore, Acari (*Pterygoplichthys pardalis*); one omnivore, Aracu (*Leporinus* sp.); and four predatory species: Piranha (*Pygocentrus nattereri*), Pirarucu (*Arapaima* sp.), Caparari (*Pseudoplatystoma fasciatum*), and Tucunaré (*Cichla ocellaris*). Sampling was performed at the end of the dry season (October to early December) and the end of the rainy season (April to July), with team members present on-site during each fishing event in all five cities.

**Table 1:** Fish Species Selected for the Study

Species	Feeding habit	Habitat
Acari (*Pterygoplichthys pardalis*)	Detritivore	Benthic: Bottoms of rivers and lakes
Aracu (*Leporinus* sp.)	Omnivoro	Benthopelagic: Deeper and darker places
Piranha (*Pygocentrus nattereri*)	Predator	Pelagics: white water rivers and lentic environments
Tucunaré (*Cichla ocellaris*)	Predator	Pelagics: blackwater rivers and lentic environments
Pirarucu (*Arapaima* sp.)	Predator	Pelagics: lakes and lentic environments
Caparari (*Pseudoplatystoma fasciatum*)	Predator	Benthic: White water river bottoms

The sampling strategy aimed to include fish species that are both highly consumed and of economic importance, selecting two representative species from each trophic guild (detritivore, omnivore, and carnivore). The objective was to obtain specimens of all six species at every sampling site; however, in some locations, certain species were unavailable for collection. The fish were measured and weighed shortly after capture, and skeletal muscle samples (fillet, i.e., boned side of the fish) were removed and immediately refrigerated. Muscle samples were stored in sealed plastic bags, kept in containers with ice, and transported to the laboratory for storage at −20 °C before being lyophilized.

### 2.2 Sample Preparation

Subsamples (approximately 1 g) were accurately weighed and digested in a mixture of 5 mL of concentrated nitric acid (TMA, Hiperpure, PanReac, Spain) and 3 mL of 30% (w/v) hydrogen peroxide (PanReac, Spain) in a microwave-assisted digestion system (Ethos Plus; Milestone, Sorisole, Italy). The digested samples were transferred to polypropylene sample tubes and diluted to 15 mL with ultrapure water according to previously described procedures and conditions.^29^

### 2.3 Toxic Element Analysis

The concentrations of the nonessential elements As, Cd, Hg, and Pb in the digested samples were determined using inductively coupled plasma mass spectrometry (ICP-MS; VG PQ Excel, Thermo Elemental, USA). A detailed description of the analytical conditions is provided elsewhere.^18^ Analytical quality control was performed throughout the study. Blank samples were processed at the same time as the test samples, and the values obtained were subtracted from the sample readings to calculate the final values. The limits of detection were calculated as three times the standard deviation of the reagent blanks and were based on the mean sample weight. In all cases, the limits of detection obtained were sufficiently low to determine all trace metals at their usual levels in the studied samples. The accuracy of the determination was evaluated by comparison with the analytical recovery of certified reference materials (fish protein DORM-3, National Research Council, Ottawa, Ontario, Canada), processed in the same way as for the samples. The good agreement between the measured and certified values (Table 2) demonstrates the high accuracy of this method, with recoveries for the certified material ranging from 91.1% (Hg) to 100.3% (Cd).

**Table 2:** Analytical Quality Program, Expressed as Mean ± Standard Deviation, Used in the Determination of Trace Elements in This Study

		Certified material reference (DORM-3)	
Metal	Detection limit (mg/kg)	Analyzed levels (mg/kg)	Certified levels (mg/kg)
As	0.006	6.62 ± 0.38	6.88 ± 0.30
Cd	0.003	0.291 ± 0.062	0.290 ± 0.020
Hg	0.001	0.348 ± 0.021	0.382 ± 0.060
Pb	0.001	0.367± 0.046	0.395 ± 0.050

### 2.4 Human Health Risk Assessment of Potentially Toxic Elements in Fish

Different methods have been used to assess the risk to human health of consuming contaminated fish.^30–33^ To determine this risk, we used the following data: the average adult body weight was 70 kg, the average daily fish consumption in the Lower Amazon was 462 g/day,^17^ and an average per capita rate of fish consumption in Brazil was 9 kg per year (24 g/person/day).^17,34^ The following health risk indicators were calculated according to their respective equations:

#### 2.4.1 Estimated Daily Intake (EDI)

1 $$EDI=\frac{C\times \mathrm{Cons}}{Bw}\times 10^{-3}$$

where *C* is the concentration of potentially toxic elements in the fish (mg/kg fresh weight), Cons is the average daily consumption of fish in the region and the national daily intake rate (462 g/day and 24 g/day), and *Bw* represents the body weight of adults (70 kg).

#### 2.4.2 Determination of the Target Hazard Quotient (THQ)

2 $$THQ=\frac{EFr\times \mathrm{EDtot}\times \mathrm{FIR}\times C}{\mathrm{RfDo}\times Bw\times \mathrm{ATn}}\times 10^{-3}$$

where EFr is the frequency of exposure (365 days/year); EDtot is the duration of exposure (70 years); FIR is the rate of food intake (g/day), with 10^–3^ as the conversion factor of the unit; *C* is the concentration of potentially toxic elements in fish (mg/kg fresh weight); RfDo is the oral reference dose for a given element (As: 0.0003, Cd: 0.001, Hg: 0.005, and Pb: 0.004 mg/kg Bw/day); *Bw* is the average adult body weight (70 kg); and ATn is the average exposure time for noncarcinogens (365 days/year × 70 years).^35^ According to USEPA guidelines,^36^ THQ values greater than 1 indicate potential noncarcinogenic health risks, suggesting adverse effects that warrant further investigation.

#### 2.4.3 Determination of the Total Target Hazard Quotient (TTHQ)

Here, the TTHQ was expressed as the arithmetic sum of the individual THQ values for each of the metals analyzed (As, Pb, Hg, and Cd). The TTHQ was interpreted using the same threshold as for the individual THQ (>1 indicates potential overall noncarcinogenic health risks).^31,36^

3 Total THQ(TTHQ)=THQ(As)+THQ(Pb)+THQ(Hg)+THQ(Cd)

#### 2.4.4 Cancer Risk (CR) for Arsenic (As) and Cadmium (Cd)

The ingestion dose exhibits a directly proportional relationship with the quantity of carcinogen ingested, with its effects being quantified through carcinogenic risk assessment (CR). The carcinogenic risk is calculated using the following equation:

4 CR=CSFo×EDI

where CSFo represents the carcinogenic slope factor or lifetime possibilities of having cancer. CSFo is 1.5 mg/kg/day for As^37^ and 0.38 mg/kg/day for Cd.^38,39^ Carcinogenic risk (CR) was classified as Negligible (below 1.0 × 10^–6^), Tolerable (between 1.0 × 10^–6^ and 1.0 × 10^–4^), and Unacceptable (above 1.0 × 10^–4^).^40^

#### 2.4.5 Total Cancer Risk (TCR)

For the assessment of combined exposure to As and Cd, the TCR was defined as the arithmetic sum of the individual CR values for each analyzed metal using eq 5:

5 TCR=CR(As)+CR(Cd)

Total cancer risk (TCR) was classified as Negligible (below 1.0 × 10^–6^), Tolerable (between 1.0 × 10^–6^ and 1.0 × 10^–4^), and Unacceptable (above 1.0 × 10^–4^). Values less than 1.0 × 10^–4^ are tolerated and do not enhance the risk of having cancer for a lifetime.^40^

## 3 Results

### 3.1 Noncarcinogenic Health Risk Assessment

The mean concentrations of toxic elements expressed in milligrams per kilogram of fresh weight for each fish species and city are presented in Table 3. Among the toxic metals studied, only Hg exceeded the maximum concentrations established in Brazilian legislation for fish intended for human consumption (0.5 mg/kg for noncarnivorous fish and 1 mg/kg for piscivorous fish).^19^

**Table 3:** Potentially Toxic Element Concentrations (Expressed in Milligrams per Kilogram of Fresh Weight) in Fish Species from the Lower Amazon

		Mean values of element concentration			
Fish species	Origin	As	Cd	Pb	Hg
Acari (*P. pardalis*)	Faro	0.01	0.001	0.001	0.04
	Itaituba	0.008	0.001	0.002	0.02
	Juruti	0.03	0.0005	0.002	0.02
	Porto Trombetas	0.004	0.00	0.002	0.1
	Santarém	0.02	0.0001	0.001	0.02
Aracu (*Leporinus* sp.)	Faro	0.002	0.0002	0.01	0.2
	Santarém	0.005	0.001	0.003	0.03
Caparari (*P. fasciatum*)	Juruti	0.003	0.0009	0.001	0.3
Piranha (*P. nattereri*)	Itaituba	0.0008	0.0005	0.002	0.53
	Juruti	0.005	0.001	0.001	0.36
Pirarucu (*Arapaima* sp.)	Santarém	0.02	0.0001	0.002	0.24
Tucunaré (*C. ocellaris*)	Faro	0.002	0.0002	0.001	0.6
	Itaituba	0.002	0.0005	0.002	0.5
	Juruti	0.01	0.0003	0.001	0.3
	Porto Trombetas	0.02	0.00	0.7	0.002
	Santarém	0.07	0.00	0.001	0.17

The results of the EDI calculation, representing the daily intake of potentially toxic elements through fish consumption in general (Table 4), indicated that the concentrations of metals were below the RfDo, except for Hg.^30^

**Table 4:** Estimated Daily Intake (EDI) for Potentially Toxic Elements Evaluated in Fish Species for Consumption in the Lower Amazon in mg/kg/day Considering the Amazon Scenario (462 g/person/day) and the Brazil Scenario (24 g/person/day)

		Amazon Scenario				Brazil Scenario			
Species	Origin	As	Cd	Hg	Pb	As	Cd	Hg	Pb
Acari (*P. pardalis*)	Faro	0.0001	0.00001	0.0002	0.00001	0.000004	0.0000005	0.00001	0.0000004
	Itaituba	0.0001	0.000003	0.0002	0.00001	0.000003	0.0000002	0.00001	0.000001
	Juruti	0.0002	0.000003	0.0001	0.00001	0.000010	0.0000002	0.00001	0.000001
	Porto Trombetas	0.00003	0.000005	0.0009	0.00001	0.000001	0.0000002	0.00005	0.000001
	Santarém	0.0002	0.000003	0.0001	0.00001	0.000008	0.0000001	0.00001	0.000000
Aracu (*Leporinus* sp.)	Faro	0.00002	0.000001	0.001	0.00005	0.000001	0.0000001	0.0001	0.000002
	Santarém	0.00003	0.00001	0.0002	0.00002	0.000002	0.0000003	0.00001	0.000001
Caparari (*P. fasciatum*)	Juruti	0.00002	0.00001	0.002	0.00001	0.000001	0.0000003	0.0001	0.000000
Piranha (*P. nattereri*)	Itaituba	0.000005	0.000003	0.003	0.00001	0.000000	0.0000002	0.0002	0.000001
	Juruti	0.00003	0.00001	0.002	0.00001	0.000002	0.0000003	0.0001	0.000000
Pirarucu (*Arapaima* sp.)	Santarém	0.0001	0.000001	0.002	0.00001	0.000006	0.0000000	0.0001	0.000001
Tucunaré (*C. ocellaris*)	Faro	0.00001	0.000001	0.004	0.00001	0.000001	0.0000001	0.0002	0.0000003
	Itaituba	0.00001	0.000003	0.003	0.00001	0.000001	0.0000002	0.0002	0.000001
	Juruti	0.0001	0.000002	0.002	0.00001	0.000003	0.0000001	0.0001	0.0000005
	Porto Trombetas	0.00001	0.000002	0.005	0.00001	0.000001	0.0000001	0.0002	0.000001
	Santarém	0.00005	0.000002	0.001	0.00001	0.000002	0.0000001	0.0001	0.0000003

The analysis of the THQ of As, Cd, Hg, and Pb is presented in Table 5. The nonessential metals As, Cd, and Pb showed values <1 for all samples captured in all scenarios. However, the level of Hg THQ in the Amazon Scenario exceeded ≥1 for almost all samples (except *P. pardalis* captured in Itaituba and Santarém). The highest Hg THQ was found in *C. ocellaris* species captured in Porto Trombetas (28.97) and Faro (23.24). Considering the Brazilian Scenario (lower fish consumption), Hg THQ was >1 for the carnivorous species *P. nattereri* (from Itaituba) and *C. ocellaris* (from Itaituba, Faro, and Porto Trombetas).

**Table 5:** Target Hazard Quotient (THQ) Calculated for Different Fish Species from the Lower Amazon Considering the Amazon Scenario (462 g/person/day) and the Brazil Scenario (24 g/person/day)^a^

		Amazon Scenario				Brazil Scenario			
Fish species	Origins	As	Cd	Hg	Pb	As	Cd	Hg	Pb
Acari (*Pterygoplichthys pardalis*)	Faro	0.25	0.01	**1.54**	0.002	0.013	0.0005	0.08	0.0001
	Itaituba	0.17	0.003	0.97	0.003	0.009	0.0002	0.05	0.0001
	Juruti	0.63	0.003	0.88	0.003	0.033	0.0002	0.05	0.0001
	Porto Trombetas	0.09	0.005	**5.65**	0.004	0.005	0.0002	0.29	0.0002
	Santarém	0.54	0.003	0.84	0.002	0.028	0.0001	0.04	0.0001
Aracu (*Leporinus* sp.)	Faro	0.05	0.001	**7.62**	0.012	0.003	0.0001	0.40	0.0006
	Santarém	0.11	0.006	**1.15**	0.005	0.006	0.0003	0.06	0.0002
Caparari (*Pseudoplatystoma fasciatum*)	Juruti	0.06	0.006	**14.24**	0.002	0.003	0.0003	0.74	0.0001
Piranha (*Pygocentrus nattereri*)	Itaituba	0.02	0.003	**21.73**	0.003	0.001	0.0002	**1.13**	0.0002
	Juruti	0.10	0.007	**14.79**	0.024	0.005	0.0003	0.77	0.0001
Pirarucu (*Arapaima* sp.)	Santarém	0.38	0.001	**10.03**	0.003	0.020	0.0000	0.52	0.0001
Tucunaré (*Cichla ocellaris*)	Faro	0.03	0.001	**23.24**	0.002	0.002	0.0001	**1.20**	0.0001
	Itaituba	0.04	0.003	**20.78**	0.003	0.002	0.0002	**1.08**	0.0001
	Juruti	0.21	0.002	**10.68**	0.002	0.011	0.0001	0.55	0.0001
	Porto Trombetas	0.04	0.002	**28.97**	0.003	0.002	0.0001	**1.50**	0.0002
	Santarém	0.15	0.002	**7.22**	0.001	0.008	0.0001	0.37	0.0001

a THQ considered an adult body weight of 70 kg and 70 years of exposure. Cells in bold indicate samples with THQ > 1.

The TTHQ values for the different fish species and sampling sites are shown in Table 6. Values >1 were found for all fish species and their respective collection areas in the Amazon Scenario, with the highest value observed for the Porto Trombetas region (29.01). However, in the Brazilian Scenario, only *P. nattereri* collected from Itaituba and *C. ocellaris* from Porto Trombetas, Faro, and Itaituba showed values >1.

**Table 6:** Total Target Hazard Quotient (TTHQ) for Metal Intake (As, Cd, Hg, and Pb) in Adults through Fish Consumption Considering the Amazon Scenario (462 g/person/day) and the Brazil Scenario (24 g/person/day)^a^

		TTHQ	
Species	Origins	Amazon Scenario	Brazil Scenario
Acari (*Pterygoplichthys pardalis*)	Faro	1.80	0.09
	Itaituba	1.15	0.06
	Juruti	1.52	0.08
	Porto Trombetas	5.75	0.30
	Santarém	1.39	0.07
Aracu (*Leporinus* sp.)	Faro	7.69	0.40
	Santarém	1.41	0.07
Caparari (*Pseudoplatystoma fasciatum*)	Juruti	14.31	0.74
Piranha (*Pygocentrus nattereri*)	Itaituba	21.76	1.13
	Juruti	14.82	0.77
Pirarucu (*Arapaima* sp.)	Santarém	10.41	0.54
Tucunaré (*Cichla ocellaris*)	Faro	23.28	1.21
	Itaituba	20.82	1.08
	Juruti	10.90	0.56
	Porto Trombetas	29.01	1.50
	Santarém	7.37	0.38

a TTHQ considered an adult body weight of 70 kg and 70 years of exposure.

### 3.2 Carcinogenic Health Risk Assessment

The results obtained for the carcinogenic risk of the metals (As and Cd) via ingestion are presented in Figure 2. In the Brazilian Scenario, the analysis of individual cancer risk (CR) for As and Cd and total cancer risk (TCR) data confirms that high fish consumption in the Amazon is a determining factor for exposure to potentially toxic elements with potential carcinogenic effects. CR values for As and Cd in the Brazil Scenario resulted in almost all data with negligible risk (≤1 × 10^–6^). However, for the Amazon Scenario, the consumption of certain fish species (Acari and Pirarucu) from western Pará cities (Faro, Juruti, and Santarém) resulted in unacceptable risk (>1 × 10^–4^) for riverside populations due to As ingestion. The TCR is presented in Figure 2. In the Brazil Scenario, TCR values remained below the risk thresholds, but for the Amazon Scenario, they exceeded the acceptable range in Acari and Pirarucu from the cities of Faro, Juruti, and Santarém, representing an unacceptable risk (Table 7).

**Table 7:** Cancer Risk (CR) for As and Cd and Total Cancer Risk (TCR) through Fish Consumption Considering the Amazon Scenario (462 g/person/day) and the Brazil Scenario (24 g/person/day)^a^

		Amazon Scenario			Brazil Scenario		
		CR			CR		
Species	Origins	As	Cd	TCR	As	Cd	TCR
Acari (*Pterygoplichthys pardalis*)	Faro	**1.1 × 10** ^**–4**^	1.4 × 10^–5^	**1.1 × 10** ^**–4**^	1.0 × 10^–5^	2.0 × 10^–7^	1.0 × 10^–5^
	Itaituba	1.0 × 10^–8^	5.0 × 10^–6^	1.3 × 10^–6^	3.9 × 10^–6^	1.0 × 10^–7^	4.0 × 10^–6^
	Juruti	**2.8 × 10** ^**–4**^	4.5 × 10^–6^	**2.8 × 10** ^**–4**^	1.0 × 10^–5^	1.0 × 10^–7^	1.0 × 10^–5^
	Porto Trombetas	4.1 × 10^–5^	6.8 × 10^–6^	4.0 × 10^–5^	2.0 × 10^–6^	1.0 × 10^–7^	2.0 × 10^–6^
	Santarém	**2.4 × 10** ^**–4**^	4.0 × 10^–6^	**2.5 × 10** ^**–4**^	1.0 × 10^–5^	1.0 × 10^–7^	1.0 × 10^–5^
Aracu (*Leporinus* sp.)	Faro	2.0 × 10^–5^	1.7 × 10^–6^	2.0 × 10^–5^	1.0 × 10^–6^	5.0 × 10^–8^	1.0 × 10^–6^
	Santarém	5.1 × 10^–5^	9.0 × 10^–6^	5.0 × 10^–5^	3.0 × 10^–6^	1.0 × 10^–7^	3.0 × 10^–6^
Caparari (*Pseudoplatystoma fasciatum*)	Juruti	2.7 × 10^–5^	8.7 × 10^–6^	3.0 × 10^–5^	1.0 × 10^–6^	1.0 × 10^–7^	2.0 × 10^–6^
Piranha (*Pygocentrus nattereri*)	Itaituba	7.0 × 10^–6^	4.5 × 10^–6^	1.1 × 10^–5^	4.0 × 10^–7^	1.0 × 10^–7^	4.0 × 10^–7^
	Juruti	4.5 × 10^–5^	8.8 × 10^–6^	5.0 × 10^–5^	2.0 × 10^–6^	1.0 × 10^–7^	2.0 × 10^–6^
Pirarucu (*Arapaima* sp.)	Santarém	**1.7 × 10** ^**–4**^	8.9 × 10^–7^	**1.7 × 10** ^**–4**^	1.0 × 10^–5^	1.0 × 10^–8^	1.0 × 10^–5^
Tucunaré (*Cichla ocellaris*)	Faro	2.0 × 10^–5^	1.7 × 10^–6^	2.0 × 10^–5^	1.0 × 10^–6^	2.0 × 10^–8^	1.0 × 10^–6^
	Itaituba	2.0 × 10^–5^	5.1 × 10^–6^	2.0 × 10^–5^	1.0 × 10^–6^	1.0 × 10^–7^	1.0 × 10^–6^
	Juruti	9.0 × 10^–5^	2.8 × 10^–6^	**0.95 × 10** ^**–4**^	5.0 × 10^–6^	4.0 × 10^–8^	5.0 × 10^–6^
	Porto Trombetas	2.0 × 10^–5^	3.4 × 10^–6^	2.0 × 10^–5^	1.0 × 10^–6^	5.0 × 10^–8^	9.5 × 10^–7^
	Santarém	7.0 × 10^–5^	2.9 × 10^–6^	7.0 × 10^–5^	4.0 × 10^–6^	4.0 × 10^–8^	3.5 × 10^–6^

a Bold cells indicate samples with unacceptable exposure risk with CR or TCR > 1 × 10^–4^.

**Figure 2:**
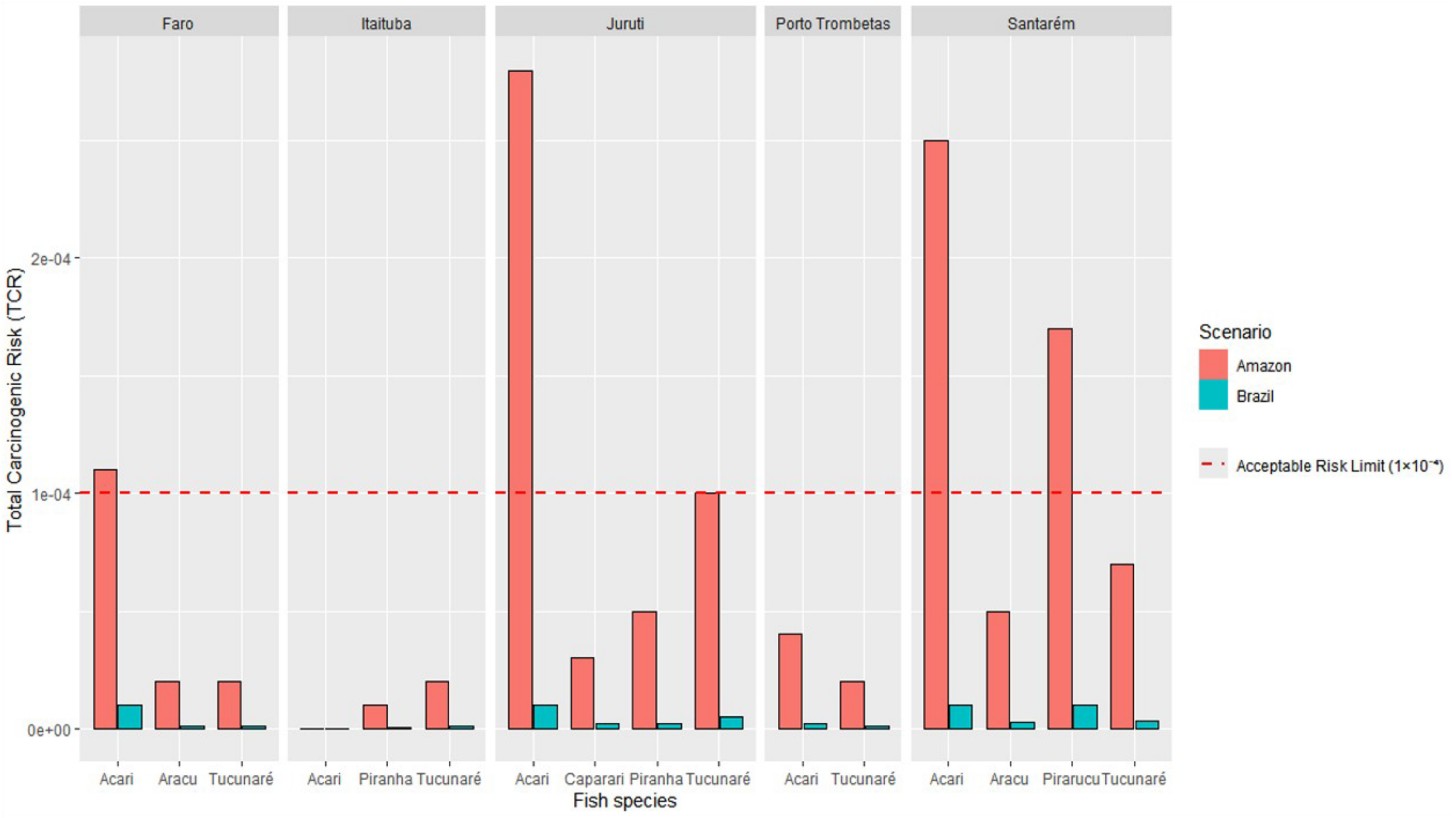
Comparison of Total Carcinogenic Risk (TCR) from fish consumption between Amazonian (462 g/person/day) and Brazilian (24 g/person/day) Scenarios. The values are presented by location and sampled species. The red dashed line indicates the acceptable risk threshold (1 × 10^–4^).

## 4 Discussion

This study provides novel insights into noncarcinogenic and carcinogenic risks from As, Cd, Hg, and Pb in fish from Western Pará, addressing a gap in research beyond Hg.^41^ Direct collaboration with fishermen ensured authenticity and traceability of sample authenticity, overcoming key limitations associated with market-purchased fish commonly used in previous studies.^42^ Our results show that national guidelines cannot be used to assess potentially toxic elements’ health risks in Amazonian populations, since fish consumption largely impacts human risk. Economic, cultural, and regional factors impose on Amazonian populations different fish intake rates, with the Amazon having one of the highest fish consumption rates in the world.^17,43–45^

Hg exhibited the highest concentrations in higher trophic level species, such as *Cichla ocellaris*, *Pygocentrus nattereri*, and *Pseudoplatystoma fasciatum*, consistent with their feeding patterns and previous data in the Amazon region. Significant element concentration variations were observed. For instance, *C. ocellaris* in Santarém had 5-fold higher As levels than in Porto Trombetas, likely due to agricultural runoff and sediment-bound As.^46^ Conversely, higher Hg levels in Itaituba and Porto Trombetas reflect gold mining impacts.^47^ *P. pardalis* in Juruti showed 10-fold higher As levels than in Porto Trombetas, possibly due to bauxite mining effluents, but lower Hg levels, indicating site-specific contamination patterns.

These findings highlight the complex interplay between anthropogenic and natural sources of elements. Santarém is located downstream of mining-impacted areas and may receive contaminated inflows; Faro lies upstream and is not directly influenced by mining-derived waters. Although both municipalities of Faro and Santarém are free from mining, their ecosystems are under pressure from agricultural activities, deforestation, fires, and illegal logging, which can adversely affect the health of the people who consume fish, particularly *C. ocellaris*, in these regions.^18,19^ When evaluating a diet rich in fish resources, it is important to consider the ability of fish to accumulate toxic metal residues, such as As, Cd, Pb, and Hg, which play an important role in the transfer of toxic elements from the diet to humans.^25,48^ Exposure to toxic elements varies widely among the fish species consumed in the Amazon region, and inhabitants who constantly consume these fish have Hg levels that indicate cumulative toxicity.^49^

The THQ, the ratio between the exposure dose and the reference dose (RfDo), represents the risk of noncarcinogenic effects. If THQ < 1, the exposure level is < RfDo, indicating that daily exposure to this level is unlikely to have negative effects during a person’s lifetime.^30,31,50^ The comparative analysis of THQ values among fish species in the Amazonian and Brazilian Scenarios reveals differences primarily influenced by distinct consumption rates (g/person/day) in each scenario. In the Amazonian Scenario, where fish consumption is high, the THQ values for Hg stand out as the most concerning, particularly in carnivorous species such as *C. ocellaris* (with THQ ranging between 7.22 and 28.97) and *P. nattereri* (with THQ between 14.79 and 21.73). These results reflect the reality of communities that heavily rely on fishing as their primary food source, exposing them to significant risks of Hg contamination, a metal known for its neurotoxic effects^49,51^ and bioaccumulative properties. In the Brazilian Scenario, where per capita consumption is considerably lower, the THQ values are drastically reduced, reflecting much lower exposure to contaminants. Besides lower exposure, high Hg THQ values are found in the Brazilian Scenario for *C. ocellaris* in Itaituba, Porto Trombetas, and Faro and for *P. nattereri* in Itaituba. However, it is important to consider that vulnerable populations, such as children, pregnant women, and the elderly, may still be subject to bioaccumulative risks, given the toxicity of Hg even at low concentrations.^52^

For the Amazonian Scenario, the THQ values of As were particularly high in the *P. pardalis* sp. collected in Juruti and Santarém, with values of 0.63 and 0.54, respectively, and were considerably lower in the Brazil Scenario. Although the calculated THQ values of As for both scenarios are below the critical values, suggesting that there is no noncarcinogenic risk for consumers, there may still be a concern regarding the consumption of fish containing As especially in the cities of Juruti and Santarém, where levels reached more than half the critical value. Considering the recent and continued anthropogenic impact in the area, As contamination could represent a health risk in the near future. This suggests that potentially toxic element contamination in the Amazon may be more concerning than in other regions of Brazil. As is a potentially toxic metalloid originating from natural (the common occurrence of arsenic sulfides along the Andes mountain range) and anthropogenic sources (the waste produced by the extraction of gold in the Amazon).^46^ As a result, there are high concentrations of As in sediments, up to 4 orders of magnitude higher than those observed in water throughout the Amazon basin, posing a risk to the local community.^46–54^

In the Brazil Scenario, all metal levels complied with the food safety standards established by the United States Environmental Protection Agency,^36^ partially due to the overall low amount of fish consumed. As and Hg HQ and THQ values in the Lower Amazon (Amazon Scenario) were higher than those reported in studies conducted in Iran, Bangladesh, Italy, Romania, the northeast Mediterranean, and New York.^55–60^ In contrast, recent studies show that, even in polluted areas, fish may present low or no human health risk for metal ingestion.^61,62^ The calculated THQ values for Hg in the cities of the Lower Amazon indicate a potential health risk associated with prolonged consumption of these fish, particularly species at higher trophic levels, whereas Cd and Pb were well below levels considered toxic to humans.^36^

To further explore the combined toxicity or interactive effects of metal toxicity and the consequences of high fish consumption in the areas considered in this study, all THQs were combined to calculate the TTHQ for the two scenarios (Amazon and Brazil), allowing a more comprehensive analysis of the risks of high fish consumption. For the Amazon Scenario, the TTHQ calculations showed that higher food chain species presented values ≥10, indicating a high potential health risk to the exposed population.^63–65^

The sources of metal contamination in the Amazon combine natural processes and anthropogenic activities.^66^ Gold and bauxite mining act as significant mobilizers of potentially toxic elements from geological formations, promoting their release into the surrounding environment through processes such as leaching, erosion, and chemical alteration of soil and groundwater.^13,67^ In the case of gold mining, considerable volumes of open-pit tailings, resulting from illegal mining operations in the Amazon, particularly in the Tapajós River basin,^68^ contain high concentrations of toxic residues of Hg, As, Cd, and Pb, which are naturally associated with sulfide-rich geological formations.^69^ Once mobilized, these metals become bioavailable for various chemical, biological, and photochemical reactions (particularly As and Hg), bioaccumulating in the trophic chain and contaminating aquatic biota, water, and sediment. Similarly, bauxite mining involves the removal of surface layers, releasing potentially toxic elements.^70^ This mobilization is exacerbated by the improper disposal of red mud, which is associated with its chemical composition and capacity to adsorb potentially toxic elements such as As, Cd, and Pb.^71^ As previously reported, due to the high levels of iron oxide in the composition of red mud,^72^ the adsorption of Hg from the natural environment^73^ and the associated impacts of bauxite mining activities^18^ may also be occurring in the adjacent regions of Juruti and Porto Trombetas. Studies have reported that soils along western and southwestern Pará constitute a significant reservoir of naturally accumulated Hg associated with fine soil particles, with Hg fixation controlled by iron and aluminum oxyhydroxides.^73,74^ These potentially toxic elements tend to become bioavailable for various chemical, biological, and photochemical reactions (particularly As and Hg), bioaccumulating in the trophic chain and contaminating all aquatic biota, water, and sediment.

Amazonian rivers play a critical role as dispersers and mobilizers of toxic elements, a process regulated by their biogeochemical characteristics and seasonal dynamics.^75^ Blackwater rivers, such as the Nhamundá (Faro), Tapajós (Itaituba), and Trombetas (Porto Trombetas) systems, characterized by high loads of dissolved organic matter and acidic pH, significantly favor the methylation and bioaccumulation of mercury (Hg) in aquatic species.^76^ In contrast, clearwater rivers of Andean origin, such as the Amazon River, which flows through the municipalities of Juruti and Santarém, exhibit higher arsenic (As) concentrations, mainly attributed to geogenic inputs from sulfated mineralization.^53^ Conversely, cadmium (Cd) and lead (Pb) concentrations remain low in both types of systems, a direct consequence of their lower solubility under neutral to acidic pH conditions.^77^ Although Hg dominates noncarcinogenic risk, the additive effects of As, Cd, and Pb, associated with toxicities such as nephrotoxicity and neurotoxicity, cannot be overlooked, especially in chronic exposures.^78,79^ The sociocultural and economic importance of fish as the primary protein source in the Amazon amplifies contaminant intake, exacerbating health risks, particularly for vulnerable populations such as children and pregnant women, where chronic Hg exposure is linked to neurological and cognitive impairments.^80,81^ The spatial variability in contamination, intensified by anthropogenic pressures and local gradients, underscores the need to consider contamination sources and rivers as vectors of toxic residues.

The concentrations found in the specimens of *P. pardalis* and *Leporinus* sp., lower trophic level species, in this study can be attributed to their feeding habits, as these species forage on river and lake bottoms where potentially toxic elements accumulate and are transferred up in the food chain.^82,83^ Our results are consistent with previous studies^84,85^ that reported high As and Hg THQ values in fish species occupying lower trophic levels.

In the Amazon region, activities such as agriculture, livestock production, deforestation, and gold and bauxite mining (particularly in western Pará) have indirectly aggravated pollution and increased heavy metal concentrations in aquatic ecosystems.^12,15,86^ Recent studies have assessed the risk of heavy metal contamination in aquatic biota and its harmful effects on the health of the population,^42,87–89^ but there are still limited reports on the health risks from the consumption of different species of fish, which are an important part of the diet of the Amazonian population. Because information concerning the risk to human health of the consumption of Acari (*P. pardalis*), Aracu (*Leporinus* sp.), Peacock Bass (*C. ocellaris*), Caparari (*P. fasciatum*), Piranha (*P. nattereri*), and Pirarucu (*Arapaima* sp.) is limited, the data obtained here were compared with those reported on other species from other locations in the Amazon biome.

The accumulation of potentially toxic elements within a given organism can be affected by species, feeding habits, age, and reproduction.^18^ In this regard, only a general comparison with previous studies can be made. It is noteworthy that Pirarucu, a large piscivorous fish that has a high commercial value and is widely consumed by tourists, is safe for consumption even when all potentially toxic elements are combined. Overall, the bioaccumulation of toxic elements in fish considered here was of the same order of magnitude or even greater than that described in other studies conducted in other areas affected by anthropogenic pollution. These areas include the Brazilian Amazon Carajás mineral province and gold mining areas in São Chico and Creporizinho, State of Pará, urban rivers of Manaus, Amazonas-AM and in the basin of the upper Paraguay River, Pantanal^42,87–89^ and in Beni River, Bolivian Amazon, and Caquetá River, Colombian Amazon.^90,91^

The integrated analysis of both individual and cumulative carcinogenic risks confirms that high fish consumption in the Amazon is a determining factor for exposure to toxic metals with carcinogenic potential. In the Amazonian Scenario, 25% of the samples presented carcinogenic risk, as per international guidelines.^92^ Arsenic was identified as the primary contributor to this risk, accounting for nearly all of the estimated value. This finding is particularly significant, given that As is classified by the International Agency for Research on Cancer (IARC) as a Group 1 carcinogen.^93^ Chronic exposure to this element is directly associated with the development of serious and potentially fatal diseases, including cancers of the bladder, lungs, kidneys, liver, and prostate, with the most common being skin cancer, Bowen’s disease, and squamous cell carcinoma, frequently observed in populations exposed to As.^93–95^ Concurrently, recent data from the Pará State Health Department (SESPA) indicate an increase in the number of skin cancer cases in the Lower Amazon region. Between 2022 and 2024, the cities of Santarém and Juruti, which exhibited the highest total cancer risk values for As, exceeding the safety threshold (see Figure 2), recorded the highest number of cases, followed by Monte Alegre, Oriximiná, and Óbidos.^96^ However, Juruti emerged as the location with the highest values of As and Cd, reinforcing the vulnerability in the Lower Amazon sub-basin. This scenario suggests that chronic As exposure, possibly linked to the high consumption of contaminated fish, particularly Acari, one of the most consumed and commercialized fish in the floodplain areas of western and southeastern Pará, along with environmental factors may contribute to the higher incidence of this neoplasm in the local population. However, under the national consumption scenario, risks remained within acceptable limits.^92^ This difference highlights that the volume of consumption is the primary risk factor, outweighing even geographical variations. It is concluded, therefore, that the traditional dietary patterns of riverside populations place them in a situation of toxicological vulnerability with elevated health risks, underscoring the urgency of public policies that integrate environmental monitoring, health surveillance, and targeted nutritional guidance for these communities.

## 5 Conclusion

This study demonstrates that fish consumption in the Amazon region represents an important pathway for human exposure to potentially toxic elements, particularly Hg and As, under high intake scenarios. The results show that, while national fish consumption patterns are associated with acceptable noncarcinogenic and carcinogenic risks, traditional Amazonian dietary habits substantially increase health risks, especially among riverine populations that rely heavily on fish as their primary protein source. The spatial variability observed among sampling sites reflects the combined influence of natural geochemical processes and anthropogenic activities, including mining, agricultural expansion, and deforestation. These findings underscore the importance of considering local environmental conditions and dietary habits when assessing health risks associated with fish consumption in the Amazon region.
